# Extravagant female sexual display in a *Megaselia* Rondani species (Diptera: Phoridae)

**DOI:** 10.3897/BDJ.3.e4368

**Published:** 2015-03-06

**Authors:** Brian V. Brown, Wendy Porras

**Affiliations:** ‡ Natural History Museum of Los Angeles County, Los Angeles, United States of America

**Keywords:** Female choice, sexual selection, tropical

## Abstract

The behavior of females of a species of *Megaselia* is described. Females perch on leaves and occasionally "dance", fluttering their wings while rapidly running on the leaf surface. During this dance, they evert bright white abdominal sacs that apparently constitute part of a visual display to attract males. The evolutionary basis of these behaviors is discussed.

## Introduction

Males of most species of flies, like most other animals, compete for females. They often have sexually-selected structural elaborations of the genitalia, or other body parts, that contact females during courtship (Eberhard 1985). In contrast, females are usually without remarkable sexually-selected characters and choose males whose particular traits stimulate them to mate and use their sperm (Eberhard 1996). Exceptions to this pattern are found in organisms in which males have some contribution to female fecundity, such as a food item, that makes them relatively more valuable to their females (Cumming 1994). In this situation, females have the unusual structural modifications and males choose their mates. Although best-studied in the dance flies (Diptera: Empididae), such role reversals also occur in the Phoridae (Sivinski 1988), and other families (e.g., Borkent and Craig 1994​).

**Figure 1.**
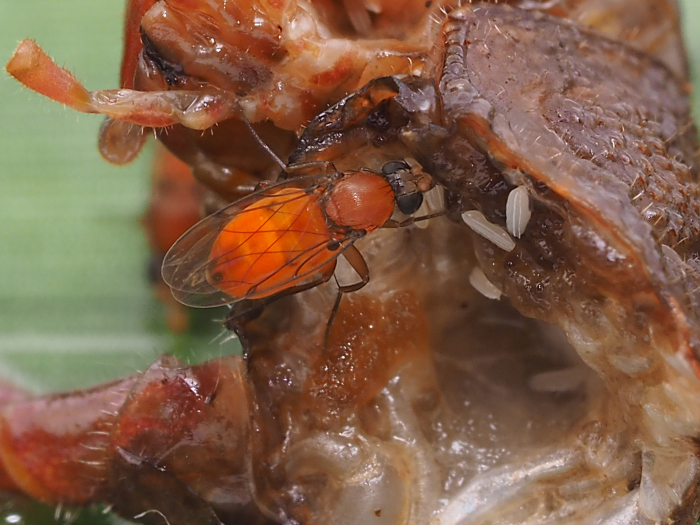
Female *Megaselia aurea* on dead cricket.

One involved genus is *Megaselia* Rondani, one of the largest radiations of Diptera, with about 1600 described and possibly 20-30,000 undescribed species (Brown 2010). With such a small percentage of species known, it is not surprising that even less is known about their behavior and lifestyles, but Sivinski (1988) did describe all-female congregations in *M. aurea* (Aldrich, 1897). This species has bright orange females (Fig. 1) that congregate on leaves of undergrowth plants, apparently to attract the less flamboyant males. Other than their bright color, there are no obvious visual cues for males, although most phorids in the subfamily Metopininae have gland openings on the dorsum of the abdomen that are thought to disperse pheromones (Disney 2003).

Our new observation is of an unidentified, but probably undescribed, species of *Megaselia* in Costa Rica. It extends our knowledge of female sexual display in the family, both in terms of behavior and associated structural modifications.

## Materials and methods

Observations were made at Zurquí de Moravia, San Jose, Costa Rica, the site of the Zurqui All Diptera Biodiversity Inventory (ZADBI) project (Brown et al. 2014). The habitat was a forested ravine at 1600 m elevation, located behind the "Restaurant La Fonda" (10.05°N, 84.02°W). Photographs and video of the displaying *Megaselia* were made by BVB 9-10 August, 2013, using a Nikon D600 camera with a Voigtländer 125 mm macro lens. Fig. 1 of *Megaselia aurea* was photographed by BVB 10 July, 2014, at La Selva Biological Station, Heredia, Costa Rica (10.43°N, 84.02°W, 50 m elevation), using an Olympus OMD-EM1 camera with a 60 mm Olympus macro lens.

The species documented here is only recognized as *Megaselia* sp. The taxonomy of *Megaselia* is based on males, whose genitalia are feature-rich, making them a superior source of features for characterization. Though we observed males visiting the females (Fig. 5​) we were unfortunately unable to capture any for species identification/description. Hopefully, as the ZADBI inventory proceeds, we will be able to link these displaying females with their conspecific males. Voucher specimens were collected and deposited in the Natural History Museum of Los Angeles County.

## Taxon treatments

### Megaselia

Rondani, 1856

#### Biology

**Figure 2.**
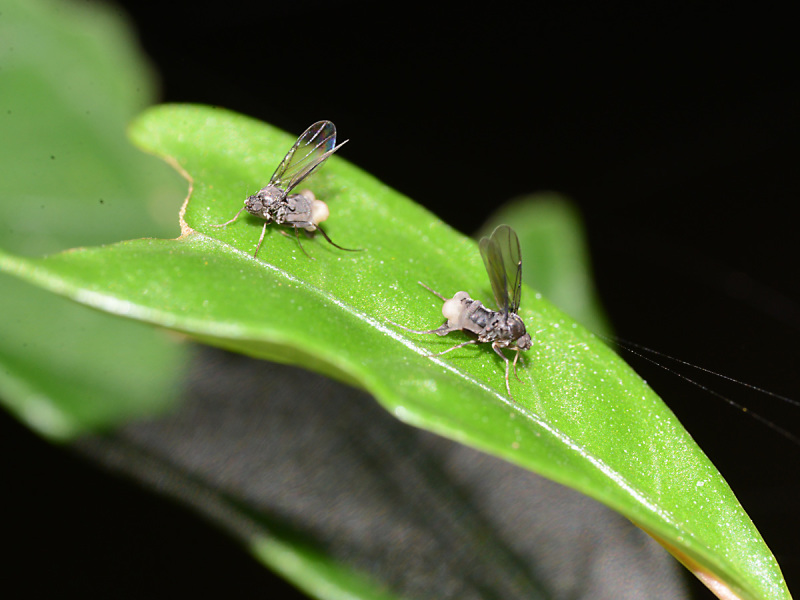
Displaying females.

**Figure 3.**
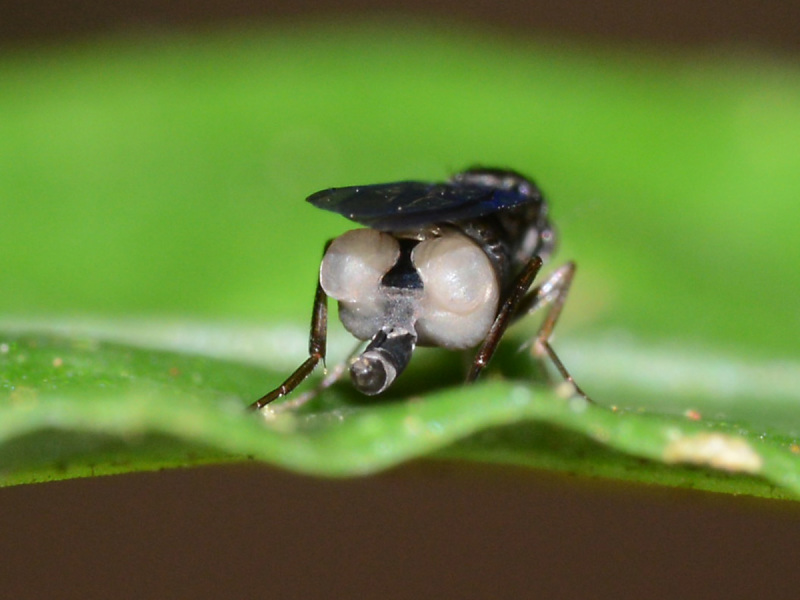
Closeup of displaying female.

**Figure 5.**
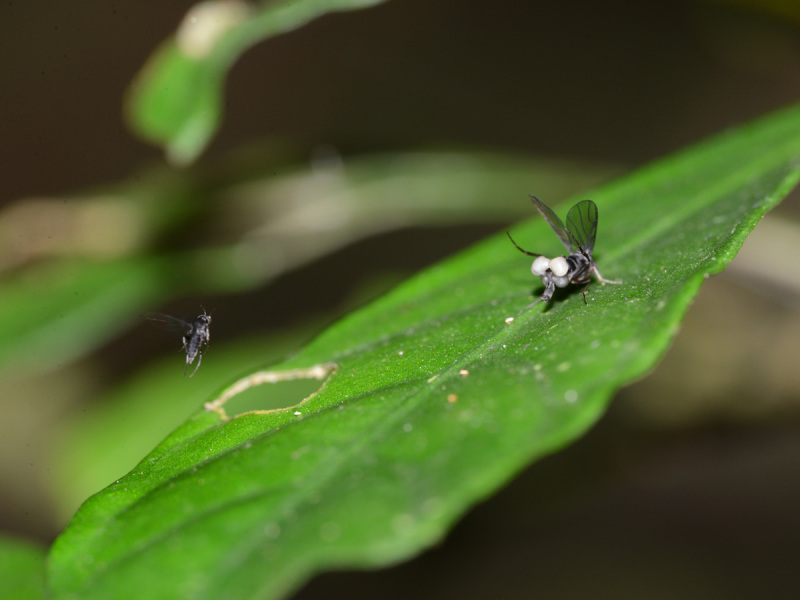
Displaying female and attracted male.

Adult females of an unidentified species of *Megaselia* Rondani were observed perching on leaves of small plants and ferns directly above a small creek. Mostly, the flies were inactive, but occasionally (when the sun came out) they began to display, by raising and fluttering their wings (Fig. 2), "dancing" (running in circles) on the leaves, and expanding the sacklike swellings in the posterior part of the abdomen (Fig. 3). Additionally, the inside surface of the hind tibia was somehow manipulated to reflect light, creating a silvery sheen (Fig. 4). The display was observed three times: once with a pair of females displaying together (Fig. 2), once with a male in attendance (Fig. 5), and once captured on video (Fig. 4). Each display lasted only a few seconds.

## Discussion

Displaying females are indicative of some sort of male contribution that overcomes the usual tendency of males to seek and compete for females. A well-known example is found in the Diptera family Empididae, in which males provide females with food during mating. Females in these interactions are frequently modified with adornments that increase the apparent size of the abdomen (Cumming 1994). These structures presumably make the female look more gravid and thus a more desirable mate. The bright white structures on the female abdomen of the *Megaselia* species probably serve the same function, making the female appear as if it is super-gravid, almost bursting with eggs. The wing-fluttering dance of these flies possibly serves to disperse pheromones that might be emitted during the display. Such chemicals, which are produced by dorsal abdominal glands found in nearly all higher metopinine phorids (Disney 2003), might be an attractant to males over longer distances.

It is unknown what, if any, nuptial gifts or resources are presented to the females, as mating was not observed, but Sivinski (1988) thought it possible that some nutrient is passed to the female in the male seminal package; this idea was discounted by Eberhard (1996), and Simmons (2001), however. Another possibility is that males are rare because of the effects of *Wolbachia* infection (R. Plowes, personal communication), making female competition for mating partners necessary. Studies in which eggs are reared to adulthood, and the resulting sex ratio recorded, are needed to investigate this possibility.

For the sacklike swellings to be deployed, it is necessary that the female has large areas free of tergites on the abdomen. For instance, in this *Megaselia*, tergite 6 is reduced to a thin band of sclerotization on the dorsum of segment 6 (Fig. 3). In the phorid taxonomic literature, such females are frequently depicted, not only in *Megaselia*, but in a wide variety of other genera (e.g., Borgmeier 1962). Furthermore, abdominal saclike structures are found in a variety of genera. They have been hypothesized to provide an increased surface area for the dissemination of pheromones (Disney 2003), but visual aspects of the displays have not been suggested previously. In addition to the bright white sacklike structures of this *Megaselia*, some species of the genera *Phalacrotophora* and *Physoptera* also have bright white on orange abdomens.

In summary, these preliminary observation draw attention to the possibility that the dominant animal mating pattern of males competing for females might be reversed in a large number of phorid fly species. Along with increasing the surface area for broadcasting pheromones, eversible female abdominal sacs might also serve as visual attractants to males, advertising falsely that females are super-fecund.

## Supplementary Material

XML Treatment for Megaselia

## Figures and Tables

**Figure 4.** Displaying female *Megaselia* sp.
